# Aspirin intake and breast cancer survival – a nation-wide study using prospectively recorded data in Sweden

**DOI:** 10.1186/1471-2407-14-391

**Published:** 2014-06-02

**Authors:** Michelle D Holmes, Henrik Olsson, Yudi Pawitan, Johanna Holm, Cecilia Lundholm, Therese M-L Andersson, Hans-Olov Adami, Johan Askling, Karin Ekström Smedby

**Affiliations:** 1 Channing Division of Network Medicine, 181 Longwood Ave, Boston, MA 02115, US; 2 Department of Epidemiology, Harvard School of Public Health, Boston, MA, US; 3 Department of Medical Epidemiology and Biostatistics, Karolinska Institutet, Stockholm, Sweden; 4 Clinical Epidemiology Unit, Department of Medicine Solna, Karolinska Institutet, Stockholm, Sweden

**Keywords:** Aspirin, Breast neoplasms, Survival, Prospective study, Sweden, Registries

## Abstract

**Background:**

Aspirin (ASA) use has been associated with improved breast cancer survival in several prospective studies.

**Methods:**

We conducted a nested case–control study of ASA use after a breast cancer diagnosis among women using Swedish National Registries. We assessed prospectively recorded ASA exposure during several different time windows following cancer diagnosis using conditional logistic regression with breast cancer death as the main outcome. Within each six-month period of follow-up, we categorized dispensed ASA doses into three groups: 0, less than 1, and 1 or more daily doses.

**Results:**

We included 27,426 women diagnosed with breast cancer between 2005 and 2009; 1,661 died of breast cancer when followed until Dec 31, 2010. There was no association between ASA use and breast cancer death when exposure was assessed either shortly after diagnosis, or 3–12 months before the end of follow-up. Only during the period 0–6 months before the end of follow-up was ASA use at least daily compared with non-use associated with a decreased risk of breast cancer death: HR (95% CI) = 0.69 (0.56-0.86). However, in the same time-frame, those using ASA less than daily had an *increased* risk of breast cancer death: HR (95% CI) = 1.43 (1.09-1.87).

**Conclusions:**

Contrary to other studies, we did not find that ASA use was associated with a lower risk of death from breast cancer, except when assessed short term with no delay to death/end of follow-up, which may reflect discontinuation of ASA during terminal illness.

## Background

In Western countries, increasingly effective adjuvant systemic treatment has entailed a gradual improvement in breast cancer survival [1]. Nevertheless, even breast cancer that is considered to have a good prognosis (node-negative, hormone –responsive) has a substantial risk of recurring within 10 years, 7-30% depending on its genetic signature [2]. Breast cancer mortality still dominates the cancer landscape in Western countries and to an increasing extent also in the developing world [3]. Hence, new and affordable therapies are urgently needed. There is accumulating pre-clinical and epidemiologic data which support a protective effect for aspirin (acetylsalicylic acid – ASA) – and perhaps some other non-steroidal anti-inflammatory drugs (NSAIDs) in breast cancer survival by an amount that rivals the benefits of currently used cancer specific therapies*.* Postulated mechanisms include the inhibition of prostaglandins which stimulate angiogenesis, inhibit apoptosis, and stimulate aromatase activity and thus increase estrogen levels; ASA may also inhibit platelet-induced adhesion of circulating tumor cells that initiate metastases [4]. Ultimately however, the mechanism is not known.

In the absence of randomized trials, large prospective observational studies remain important to advance this field of research. To this end, we used unique Swedish population-based registers allowing a prospective nation-wide study with drug intake ascertained through a prescription register. We hypothesized that women with breast cancer who took ASA would experience a lower risk of death from breast cancer compared to similar women not taking ASA.

## Methods

### Study cohort

Using the Swedish National Cancer Register, we identified 33,697 female patients with a first incident breast cancer diagnosis between April 1, 2005 and December 31, 2009. We excluded 6,244 patients who had a record of another cancer diagnosis (except non-melanoma skin cancer) prior to breast cancer, 22 cases diagnosed at autopsy, and 5 individuals with erroneous coding of dates of last follow up. Reporting of cancers by clinicians and pathologists has been required by Swedish law since 1958, and the completeness of the Cancer Registry is now approaching 100% for breast cancer. Using the National Registration Numbers assigned to all Swedish residents, the cohort was further linked with other nation-wide registers including the Prescribed drug Registry, the Cause of death Registry, the Patient Registry and the LISA (longitudinal integration database for health insurance and labor market studies) registry including information on highest achieved educational level (≤9 years, 10–12 years, >12 years) [5].

Linkage to the Population Registry allowed us to censor the 44 women who were lost to follow-up because they moved out of the country. Linkage to the death registry allowed us to achieve virtually complete follow-up with regard to vital status and to ascertain date of death as well as cause of death up to Dec 31, 2010.

The study was approved by the Regional Ethics Committee, Karolinska Institutet, Stockholm Sweden (2007/1335-31/4). No patient consent was needed.

### Study design

Within the final cohort of 27,424 patients with a first incident breast cancer diagnosed during the study period, we used a nested case–control design to investigate the association between the dispensing of ASA at different time intervals after breast cancer diagnosis and risk of breast cancer death. In the main analysis, cases were all individuals in the cohort experiencing death due to breast cancer during the study period starting from 3 months following breast cancer diagnosis. We did not consider exposure or outcome events during the first three months after diagnosis since most women are expected to use pain killers including ASA immediately following surgery. Death due to breast cancer was defined as having breast cancer as the main cause of death.

For each case we randomly selected 2 controls via risk-set sampling using time since diagnosis as the time scale. Additionally, cases and controls were matched on age and calendar year of breast cancer diagnosis. Since we sampled controls at the time of the event of the case, we took drop outs and other deaths into account*.* The end of follow-up for each matched risk set was considered to be the time of death for the case. In analyses stratified by stage at breast cancer diagnosis (stage I, II, III-IV), new controls were sampled to the cases in each stratum and additionally matched on breast cancer stage. In a secondary analysis, cases were defined as breast cancer patients who died of non-breast-cancer related causes, and controls were sampled from within the cohort using a similar procedure as described above.

### Classification of drug intake

The Swedish Prescription registry has recorded all prescriptions dispensed at Swedish pharmacies prospectively beginning from 1^st^ of July 2005 [6]. In the prescription registry, we ascertained any dispensing of prescribed low dose ASA during the entire period of follow-up. Our definition of dispensed ASA was limited to daily doses of 75 or 160 mg (ATC codes B01AC06, 30 and 56), as these doses represent 90% of all ASA forms sold nationally, and are available only by prescription.

We classified drug intake according to the following principles: within each six-month period of follow-up, we added up the total number of dispensed ASA and categorized drug intake into three different groups; unexposed individuals (0 daily doses), partially exposed individuals (less than 1 daily dose) and fully exposed individuals (1 or more daily doses of either 75 or 160 mg). To account for possible variation in the dispensing of drugs during follow-up, exposure was assessed during different time windows; 3–9 months following diagnosis, 6–12 months before end of follow-up, 3–9 months before end of follow-up and, 0–6 months before end of follow-up. Furthermore, we examined cumulative exposure as the percentage of follow-up time as exposed in three different analyses. The date of entry (i.e. 3 months following diagnosis) was used as starting point in all three analyses, and the proportion of follow-up time as exposed was summed up to 6 months before end of follow-up, 3 months before end of follow-up, or up to the end of follow-up, respectively.

Non-aspirin NSAIDs may also affect breast cancer survival, and NSAID use may correlate with ASA use. Therefore, since NSAID use may confound the association tested, we assessed NSAID dispensings (ATC codes starting with M01A) in a similar fashion to ASA, as described above, in order to be able to adjust for NSAID use as a covariate (yes/no in each time window investigated).

### Ascertainment of co-morbidity

The Swedish National Board of Health and Welfare has compiled data on individual hospital discharges in its Patient registry which has had nation-wide coverage since 1987 as previously described [7]. Besides national registration number, each record contains medical data including diagnosis at discharge according to the International Classification of Diseases X^th^ Revision. Since 2001, this register also records visits in specialized outpatient care. Because of concern that underlying diseases might confound an association between drug intake and breast cancer survival, we linked the entire study cohort to the Patient registry for the period 1987 to 2009. Co-morbidity was assessed in two major groups: diseases for which ASA use is recommended (cardiovascular, inflammatory and cerebrovascular disorders) and diseases where ASA may be counter-indicated (thromboembolism, peptic ulcer disease, chronic liver failure, and asthma). Since the Patient Registry is confined to records of hospital admissions and/or specialized outpatient visits and not visits to the general practitioner, we consider the comorbidity assessment to represent severe disorders.

### Statistical analysis

We analyzed the association between low-dose ASA and risk of breast cancer death, using conditional logistic regression, with one analysis for each exposure window investigated. All models were adjusted for co-morbid diseases and educational level as a proxy for socioeconomic status. For each analysis the co-morbid diseases status was evaluated before the start of each exposure window, thus allowing co-morbid status to vary between analyses. For analysis per each time window, only cases (and the respective controls) that were followed-up long enough to experience the whole exposure window were included. For example, to be included in the analysis of exposure 3–9 months before end of follow-up the case had to survive at least 9 months from entry. Since we use a nested case–control design with risk-set sampling of controls, we were in fact measuring the same quantity as in a time-to-event analysis. By sampling controls among those at risk at each time of an event, we have controlled for follow-up time, and the estimated odds ratios from the conditional logistic regression were therefore regarded and reported as hazard ratios [8].

## Results and discussion

### Results

**Figure 1:**
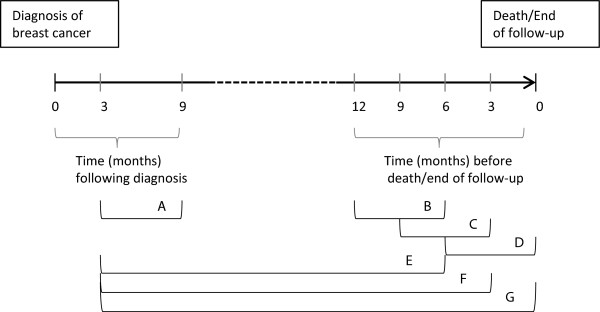
Overview of time periods of exposure assessment.

Table 1 shows the characteristics of women diagnosed with incident breast cancer during the study period. Of the 27,426 women diagnosed, 1,661 of them died of breast cancer during a median follow-up of 2.57 years. In addition, 1,371 died of other causes. A relatively small proportion (12.6%) had severe co-morbid conditions associated with recommendations to use ASA or not. Figure 1 presents an overview of the time periods of exposure assessment that are shown in the subsequent tables. Each method of exposure assessment is labeled with the letters A – G.

**Table 1:** Characteristics of women with incident breast cancer in Sweden April 1, 2005 to December 31, 2009

**Characteristic**	**Breast cancer patients N (%)**	**Breast cancer deaths N (%)**
Total number	27426	1661 (6.1)
Follow-up time, years, median (range)	2.57 (0–5.25)	1.48 (0–4.50)
**Age at diagnosis, years**		
20-29	104 (0.4)	11 (10.6)
30-39	1042 (3.8)	73 (7.0)
40-49	4098 (14.9)	175 (4.3)
50-59	6259 (22.8)	283 (4.5)
60-69	7869 (28.7)	329 (4.2)
70-79	4441 (16.2)	323 (7.3)
80-89	3037 (11.1)	370 (12.2)
90≤	576 (2.1)	97 (16.8)
Median (range)	62 (20–102)	68 (21–102)
**Calendar year of diagnosis**		
2005	4267 (15.6)	451 (10.6)
2006	5769 (21.0)	477 (8.3)
2007	5721 (20.9)	359 (6.3)
2008	5829 (21.3)	250 (4.3)
2009	5840 (21.3)	124 (2.1)
**Stage**		
I	12645 (46.1)	158 (9.5)
II	9715 (35.4)	670 (40.3)
III	1381 (5.0)	316 (19.0)
IV	438 (1.7)	227 (13.7)
Missing	3247 (11.8)	290 (17.5)
**Severe comorbidity**		
Disorders associated, with use of ASA*	2334 (8.5)	362 (15.5)
Disorders associated, with decreased use of ASA**	1116 (4.1)	182 (16.3)

*Hospitalizations or specialized outpatient visits for chronic inflammatory, cardiovascular or cerebrovascular disorders.

**Hospitalizations or specialized outpatient visits for asthma, peptic ulcers, chronic liver disease or venuous thromboembolism.

Overall survival in the group was 94% at 2 years and 83% at 5 years after diagnosis. (Additional file 1: Figure S1) Approximately 10% of the cases and controls (10.8% among the cases and 10.2% among controls) changed ASA exposure category from baseline to the next-to-last time window assessed (9–3 months before end of follow-up). Comparing baseline to the last time window (6–0 months before end of follow-up), 12.8% of the cases and 10.8% among the controls had changed exposure status.

Table 2 shows the relationship between ASA intake and breast cancer death, with ASA intake assessed at varying 6-month periods between diagnosis and death/end of follow-up. When we excluded exposure during the first 3 months after diagnosis (presumed to be the post-operative period), the baseline exposure assessment was considered to be 3 to 9 months after diagnosis (Exposure Period A). Exposure Periods B, C, and D were 12 to 6 months, 9 to 3 months, and 6 to 0 months before death/end of follow-up. Table 2 contains two types of models. Model 1 was adjusted only for age and calendar year at diagnosis, and time since diagnosis. Model 2 was additionally adjusted for co-morbid disease and education level. We found a modest change in the hazard ratios (HR) between models 1 and 2, indicating a modest amount of confounding by disease co-morbidity and education.

**Table 2:** Relative risk (HRs and 95% Confidence Intervals, CI) of breast cancer death in association with dispensed ASA dose at different time periods following breast cancer diagnosis using a nested case–control design

**Period**	**ASA dose**	**Cases**	**Controls**	**Model 1**	**Model 2**
				**HR (95% CI)**	**HR (95% CI)**
**A**	**- Baseline (3 to 9 months after diagnosis)**				
		(n = 1521)	(n = 3042)		
	0	1235 (81.2)	2481 (81.6)	1.00	1.00
	<1 daily dose	78 (5.1)	146 (4.8)	1.08 (0.81; 1.44)	1.13 (0.84; 1.52)
	≥ 1 daily dose	208 (13.7)	415 (13.6)	1.01 (0.84; 1.23)	0.97 (0.79; 1.18)
**B**	**- 12 to 6 months before end of follow-up**				
		(n = 1211)	(n = 2422)		
	0	964 (79.6)	1961 (81.0)	1.00	1.00
	<1 daily dose	72 (5.9)	138 (5.7)	1.08 (0.80; 1.46)	1.05 (0.77; 1.44)
	≥ 1 daily dose	175 (14.5)	323 (13.3)	1.12 (0.90; 1.38)	1.02 (0.81; 1.28)
**C**	**- 9 to 3 months before end of follow-up**				
		(n = 1380)	(n = 2760)		
	0	1102 (79.9)	2216 (80.3)	1.00	1.00
	<1 daily dose	81 (5.9)	152 (5.5)	1.07 (0.81; 1.42)	1.00 (0.74; 1.34)
	≥ 1 daily dose	197 (14.3)	392 (14.2)	1.01 (0.83; 1.24)	0.93 (0.75; 1.15)
**D**	**- 6 to 0 months before end of follow-up**				
		(n = 1521)	(n = 3042)		
	0	1220 (80.2)	2420 (79.6)	1.00	1.00
	<1 daily dose	120 (7.9)	159 (5.2)	1.49 (1.16; 1.92)	1.43 (1.09; 1.87)
	≥ 1 daily dose	181 (11.9)	463 (15.2)	0.77 (0.63; 0.93)	0.69 (0.56; 0.86)

Model 1: Logistic regression model with adjustment for the matching factors age at diagnosis, calendar year of diagnosis, and time since diagnosis.

Model 2: Additional adjustment for comorbidity (in groups of disorders associated with increased or decreased use of ASA) and highest obtained educational level (≤9 years, 10–12 years, >12 years).

There was no association between ASA use and breast cancer death in Exposure Periods A, B, and C. For Exposure Period D only (6 to 0 months before end of follow-up), was there a decreased risk of breast cancer death among those using ASA at least daily compared to non-users; HR (95% CI) = 0.69 (0.56-0.86). However, we found no evidence of a dose–response association in this time-window, as those using ASA less than daily had an *increased* risk of breast cancer death: HR (95% CI) = 1.43 (1.09-1.87).

In Table 3 we show the results of assessing cumulative ASA intake beginning with the baseline period. We assessed the result of ending this cumulative ASA exposure at varying times before the end of follow-up. In Exposure Periods E, F, and G we ended assessment of cumulative ASA exposure 6 months, 3 months, and 0 months before the end of follow-up, respectively. ASA intake was assessed as a percentage of the follow-up time, and was categorized as 0%, >0 – 25%, >25 – 50%, >50-75%, and >75% of the time. Models were the same as in Table 2. When assessed cumulatively, there was no association between ASA intake and risk of breast cancer death regardless of when exposure assessment ended. In addition, there was no evidence of a dose response with increasing percentages of time using ASA.

**Table 3:** Relative risk (HRs and 95% Confidence Intervals, CI) of breast cancer death in association with cumulative ASA dispensing defined as percent of time as exposed from breast cancer diagnosis to end of follow-up

**Period**	**ASA dose**	**Cases**	**Controls**	**Model 1**	**Model 2**
				**HR (95% CI)**	**HR (95% CI)**
**E**	**- Up to 6 months before end of follow-up**				
		(n = 1521)	(n = 3042)		
	0%	1206 (79.3)	2439 (80.2)	1.00	1.00
	>0 – 25%	23 (1.5)	40 (1.3)	1.18 (0.69; 1.99)	1.28 (0.74; 2.21)
	>25 – 50%	22 (1.5)	44 (1.4)	1.02 (0.60; 1.74)	1.18 (0.68; 2.04)
	>50 – 75%	29 (1.9)	63 (2.1)	0.94 (0.60; 1.47)	0.99 (0.63; 1.58)
	>75%	241 (15.8)	456 (15.0)	1.08 (0.90; 1.30)	1.05 (0.87; 1.28)
**F**	**- Up to 3 months before end of follow-up**				
		(n = 1661)	(n = 3322)		
	0%	1298 (78.1)	2622 (78.9)	1.00	1.00
	>0 – 25%	31 (1.9)	48 (1.4)	1.32 (0.83; 2.10)	1.47 (0.91; 2.38)
	>25 – 50%	26 (1.6)	60 (1.8)	0.88 (0.55; 1.41)	0.98 (0.60; 1.59)
	>50 – 75%	41 (2.4)	74 (2.2)	1.13 (0.76; 1.66)	1.18 (0.80; 1.76)
	>75%	265 (16.0)	518 (15.5)	1.04 (0.88; 1.24)	1.00 (0.83; 1.20)
**G**	**- Up to end of follow-up**				
		(n = 1661)	(n = 3322)		
	0%	1266 (76.2)	2572 (77.4)	1.00	1.00
	>0 – 25%	43 (2.6)	57 (1.7)	1.56 (1.04; 2.34)	1.65 (1.08; 2.53)
	>25 – 50%	34 (2.0)	64 (1.9)	1.09 (0.71; 1.68)	1.20 (0.77; 1.85)
	>50 – 75%	54 (3.3)	89 (2.7)	1.25 (0.88; 1.78)	1.27 (0.89; 1.82)
	>75%	264 (15.9)	540 (16.3)	1.00 (0.84; 1.19)	0.96 (0.80; 1.16)

Model 1: Logistic regression model with adjustment for the matching factors age at diagnosis, calendar year of diagnosis, and time since diagnosis.

Model 2: Additional adjustment for comorbidity (in groups of disorders associated with increased or decreased use of ASA) and highest obtained educational level (≤9 years, 10–12 years, >12 year).

The analyses in Tables 2 and 3 were repeated with additional adjustment for stage at diagnosis (4 stages); there was no material change in the results (data not shown). In addition, the analyses in Tables 2 and 3 were also repeated with additional adjustment for use of other NSAIDs; again results did not materially change (data not shown). The hazard for breast cancer death for non-ASA NSAID use (compared to non-use) in models adjusted for all factors (including ASA use) was greater than one and increased as the end of follow-up was approached (Additional file 2: Table S3).

In secondary analyses, we assessed whether results differed by stage at diagnosis. We repeated the analysis in Table 2, stratified by the categories Stage I, Stage II, and Stages III + IV (Additional file 2: Table S1). The results did not differ substantially from the non-stratified analyses (those in Table 2).

We assessed whether ASA intake was associated with non-breast cancer causes of death in an analysis similar to that of Table 2 (Additional file 2: Table S2). Compared to non-use, intake of ASA was associated with increased risk of other causes of death. This was particularly true for the earlier Exposure Periods A, B, and C and less so for Exposure Period D, with evidence of a dose–response in periods A, B and C. For example, for the baseline (Exposure Period A), the HR (95% CI) for no intake, <1 daily dose, and >1 daily dose were 1.00 (reference), 1.23 (0.96-1.58), and 1.35 (1.14-1.60), respectively.

### Discussion

In this nested case–control study using linked prospectively recorded Swedish cancer registry, death registry, and national pharmacy data, we found little evidence that ASA intake among women diagnosed with breast cancer reduces risk of breast cancer death. After assessing ASA exposure multiple different ways, we found that only when ASA intake was assessed short term without any delay (i.e., in the six-month-period preceding death/end of follow-up) was it associated with a reduced risk of breast cancer death similar in magnitude to that reported in most other studies assessing ASA and/or NSAIDs [9-13]. This result is concerning for the possibility of confounding by indication: women who are ill from metastatic cancer may forgo ASA prescribed primarily to prevent heart disease, likely considered less relevant as they approach death from breast cancer. It may also be that our exposure ascertainment fails when patients are admitted to hospices or other forms of terminal care where drugs use will not be recorded in the Prescribed Drug Registry. Also, we found an increasing hazard of breast cancer death with non-ASA NSAID use as the end of follow-up approached. This result is best explained by women using more NSAIDs for pain control as they approached death, i.e., confounding by indication.

Limitations of our study include the following: ASA is available over-the-counter in Sweden as in all countries. Since we relied on pharmacy records, we undoubtedly misclassified ASA use as many categorized as non-users by pharmacy records were likely users. However, low-dose ASA (for example to prevent heart disease) constitutes close to 90% of all ASA sold in Sweden, and low-dose forms are only available through prescription [14]. With the data at hand, we were only able to classify dose based on the amount of tablets dispensed in relation to recommended daily dose, and we could not distinguish between dispensings of tablets of 75 mg or 160 mg ASA (the two options of low-dose ASA that exist in Sweden). In addition, we regret not having access to additional clinical data on breast cancer characteristics and treatment for further adjustment, although we do not think that these factors necessarily represent strong potential confounders of ASA use and breast cancer prognosis beyond stage.

We must consider why our results differ from other prospective studies of the same question. Of the four published studies, The Iowa Women’s Health Study reported a RR (95% CI) of breast-cancer death, 0.53 (0.30-0.93) for women with breast cancer using ASA compared to nonusers [10], and the Nurses’ Health Study reported a similar result: 0.51 (0.41-0.65) [11]. The Life After Cancer Epidemiology (LACE) cohort reported no association with ASA (RR (95% CI) = 1.09 (0.74-1.61)) using recurrence as the outcome, but a significantly lower recurrence for current NSAID intake, RR (95% CI) = 0.56 (0.33-0.95) [9]. A New York based cohort of women with breast cancer reported no association of pre-diagnostic ASA use with breast cancer death, RR (95% CI) = 0.82 (0.54-1.24) [15].

Firstly, misclassification of ASA use mentioned in the limitations above (over-the-counter ASA users misclassified as non-users, and our inability to distinguish between doses of ASA) would tend to underestimate any ASA effect.

Secondly, follow-up time in the two previous studies null for ASA intake [9,15], and in our present study, was considerably less than that for the two studies which found an ASA advantage [10,11]; mean 2.5 years for LACE, [9], median 2.6 years for the current study, mean 7.3 years for the New York cohort [15], and mean 8.3 years for the Iowa Women’s Health Study [10], and maximum 30 years for the Nurses’ Health Study [11] which may explain these differing results. Another aspect of potential importance for the discrepant observations is varying time periods of exposure assessment. In the present study, we used the nested case–control design to closely examine the effect of timing of ASA use. Only in the last period of follow-up did we observe a reduced risk among daily users, possibly explained by decreased intake due to terminal illness or in-hospital drug administration, although a true effect cannot be excluded. Along these lines, in one of the studies reporting a reduced risk with ASA, the inverse association was observed with current but not past use [11]. However, it should be noted that the association with current use in that study remained in analyses of risk of cancer recurrence, likely less affected by changes towards the end of life.

Our lack of finding a cumulative dose or duration effect was similar to one other published study [11]. The fact that as much as 55% of women who died in our study, died of breast cancer could be expected due to the short follow-up (median 2.6 years), as the peak of recurrences occurs within the first 5 years after diagnosis, particularly among those whose tumors are hormone receptor negative [16].

Recently published data from randomized trials provide intriguing evidence for the effect of ASA on cancer recurrence. Data was pooled from 5 large United Kingdom trials of ASA to prevent vascular disease and examined for the effect of ASA on cancer metastases presenting during the trials or after they ended. Those subjects treated with ASA had a substantially reduced risk of metastatic adenocarcinoma (of any site) (RR = 0.52, 95% CI = 0.35-0.75). Although it was difficult to examine individual cancer sites because of small numbers, there was a suggestion of reduced case-fatality for breast cancer (RR = 0.16, 95% CI = 0.02-1.19) [12].

We found an increased risk of non-breast cancer death for ASA users. This makes sense because ASA users are most likely taking it as secondary prevention for cardiovascular disease. It may also indicate that we could not fully adjust for co-morbidity. However, the extent to which co-morbidity is associated with breast cancer survival, potentially leading to residual confounding in our data, is unclear. We also found an increased risk of breast cancer death among patients taking less than 1 dose/day on average, during the 6 months prior to death (or corresponding date for matched controls). However, this may be due to reverse causation, as terminally ill breast cancer patients might stop secondary prevention for cardiovascular disease or be admitted to hospital for terminal care, and consequently their medication would not be recorded in the Swedish prescription registry, since the register does not cover drugs distributed in hospitals.

## Conclusion

In conclusion, in this population-based Swedish register study, we did not find that use of ASA among women with breast cancer was associated with a lower risk of death from breast cancer. This is contrary to results from some but not all other prospective cohort studies. We found that ASA intake was associated with a reduced risk of breast cancer death only when it was assessed short term with no delay to death or end of follow-up, and also that non-breast cancer deaths were higher among ASA users. Because of these two findings, we speculate that the phenomenon of confounding by indication may be contributing to the conflicting results from prospective studies.

We suggest a randomized trial of ASA in women with breast cancer specifically with breast cancer death as the outcome, for efficiency limited to women with stages II and III tumors at higher risk of recurrence. Because of the risk of serious gastrointestinal bleeding or hemorrhagic stroke, ASA is currently recommended to prevent heart disease only among those women considered to be high risk (previous myocardial infarction or stroke, with angina or coronary artery stent or revascularization, or with diabetes over age 60). Such women for whom ASA is indicated would ethically have to be excluded from a trial of ASA for breast cancer survival. A randomized trial will be the only way to sort out the issues of confounding by indication, balance the risk of mortality from bleeding versus a potential benefit on breast cancer survival, and determine a causal relationship between breast cancer prognosis and ASA.

## Abbreviations

ASA: Aspirin, acetylsalicylic acid; CI: Confidence interval; HR: Hazard ratio; LACE: Life after cancer epidemiology; LISA: Longitudinal integration database for health insurance and labor market studies; NSAID: Non-steroidal anti-inflammatory drug; OR: Odds ratio.

## Competing interests

The authors declare that they have no competing interests.

## Authors’ contributions

Each author has participated sufficiently in the work to take public responsibility for appropriate portions of the content. MDH contributed to the conception and design of the study, the analysis and interpretation of the data, drafting the manuscript, revised the manuscript critically for intellectual content, and approved the final manuscript. HO contributed to the conception and design of the study, the analysis and interpretation of the data, revised the manuscript critically for intellectual content, and approved the final manuscript. YP contributed to the analysis and interpretation of the data, revised the manuscript critically for intellectual content, and approved the final manuscript. JH contributed to the analysis and interpretation of the data, revised the manuscript critically for intellectual content, and approved the final manuscript. CL contributed to the analysis and interpretation of the data, revised the manuscript critically for intellectual content, and approved the final manuscript. TM-LA contributed to the analysis and interpretation of the data, revised the manuscript critically for intellectual content, and approved the final manuscript. H-OA contributed to the conception and design of the study, the analysis and interpretation of the data, revised the manuscript critically for intellectual content, and approved the final manuscript. JA contributed to the conception and design of the study, the analysis and interpretation of the data, drafting the manuscript, revised the manuscript critically for intellectual content, and approved the final manuscript. KES contributed to the conception and design of the study, acquisition of the data, the analysis and interpretation of the data, drafting the manuscript, revised the manuscript critically for intellectual content, and approved the final manuscript.

## Authors information

Johan Askling and Karin Ekström Smedby shared senior author position.

## Pre-publication history

The pre-publication history for this paper can be accessed here:

http://www.biomedcentral.com/1471-2407/14/391/prepub

## Supplementary Material

Additional file 1: Figure S1 Kaplan-Meier estimates of all-cause survival. Click here for file

Additional file 2: Table S1 Relative risk (HRs and 95% Confidence Intervals, CI) of breast cancer death in association with dispensed ASA dose at different time periods following breast cancer diagnosis stratified by cancer stage at diagnosis. **Table S2.** Relative risk (HRs and 95% Confidence Intervals, CI) of other causes of death (non-breast cancer causes) in association with dispensed ASA dose at different time periods following breast cancer diagnosis using a nested case–control design. **Table S3.** Relative risk (HRs and 95% Confidence Intervals, CI) of breast cancer death in association with dispensed NSAID dose at different time periods following breast cancer diagnosis using a nested case–control design. Click here for file
