# Supplementary figures and images for: Aspirin intake and breast cancer survival – a nation-wide study using prospectively recorded data in Sweden
Source: BMC Cancer. 2014 Jun 2;14:391. doi: 10.1186/1471-2407-14-391 (PMC4065077; doi:10.1186/1471-2407-14-391)

## Slide 1
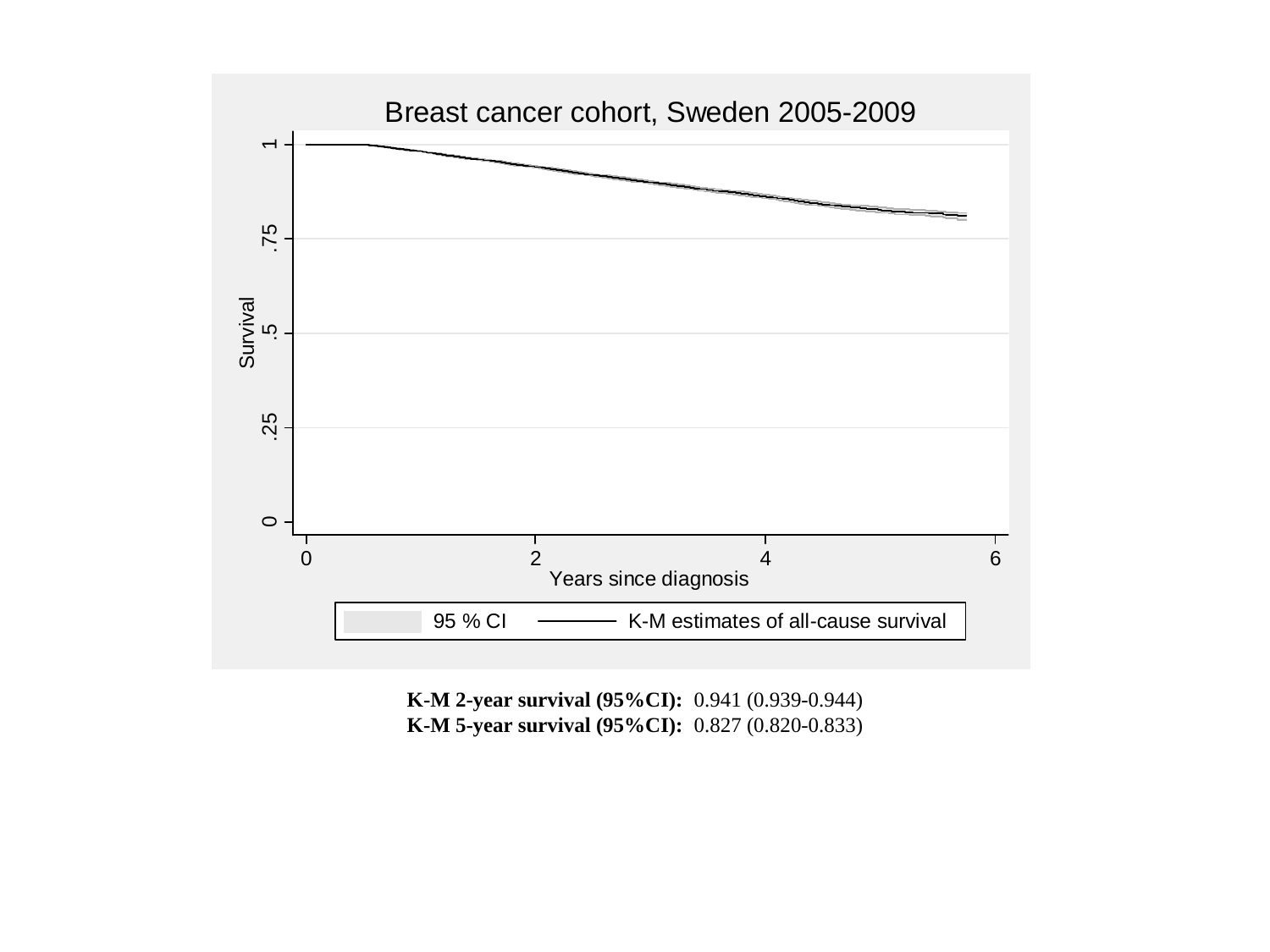

K-M 2-year survival (95%CI): 0.941 (0.939-0.944)
K-M 5-year survival (95%CI): 0.827 (0.820-0.833)

Supplement: Additional file 1: Figure S1 — Kaplan-Meier estimates of all-cause survival. [file 1471-2407-14-391-S1.pptx]
