# Supplementary material for: Aspirin intake and breast cancer survival – a nation-wide study using prospectively recorded data in Sweden
Source: BMC Cancer. 2014 Jun 2;14:391. doi: 10.1186/1471-2407-14-391 (PMC4065077; doi:10.1186/1471-2407-14-391)
Supplement: Additional file 2: Table S1 — Relative risk (HRs and 95% Confidence Intervals, CI) of breast cancer death in association with dispensed ASA dose at different time periods following breast cancer diagnosis stratified by cancer stage at diagnosis. Table S2. Relative risk (HRs and 95% Confidence Intervals, CI) of other causes of death (non-breast cancer causes) in association with dispensed ASA dose at different time periods following breast cancer diagnosis using a nested case–control design. Table S3. Relative risk (HRs and 95% Confidence Intervals, CI) of breast cancer death in association with dispensed NSAID dose at different time periods following breast cancer diagnosis using a nested case–control design. [file 1471-2407-14-391-S2.pdf]

Supplementary Table 1. Relative risk (HRs and 95% Confidence Intervals, CI) of breast cancer death in association with dispensed ASA dose at different time periods following breast cancer diagnosis stratified by cancer stage at diagnosis.

Period	ASA dose	Stage I		Stage II		Stage III and IV	
		**Ca/co**	**HR (95% CI)**	**Ca/co**	**HR (95% CI)**	**Ca/co**	**HR (95% CI)**
**A**	**- baseline (3 to 9 months after diagnosis)**						
		(151/302)		(621/1242)		(478/955)	
	0	128/244	1.00	493/992	1.00	398/794	1.00
	<1 daily dose	4/14	0.58 (0.16; 2.02)	33/62	1.07 (0.68; 1.69)	28/52	1.05 (0.63; 1.73)
	≥ 1 daily dose	19/44	0.88 (0.45; 1.72)	95/188	1.03 (0.76; 1.39)	52/109	0.97 (0.66; 1.43)
**B**	**- 12 to 6 months before end of follow-up**						
		(124/248)		(507/1014)		(352/703)	
	0	104/203	1.00	401/811	1.00	282/576	1.00
	<1 daily dose	3/8	0.79 (0.19; 3.30)	27/51	1.12 (0.68; 1.84)	30/31	2.19 (1.27; 3.78)
	≥ 1 daily dose	17/37	0.89 (0.45; 1.79)	79/152	1.01 (0.72; 1.40)	40/96	0.86 (0.56; 1.32)
**C**	**- 9 to 3 months before end of follow-up**						
		(142/284)		(573/1146)		(418/835)	
	0	116/228	1.00	452/901	1.00	342/689	1.00
	<1 daily dose	7/17	0.85 (0.33; 2.19)	33/67	0.87 (0.55; 1.38)	27/37	1.59 (0.93; 2.71)
	≥ 1 daily dose	19/39	0.96 (0.50; 1.84)	88/178	0.97 (0.70; 1.33)	49/109	0.86 (0.58; 1.29)
**D**	**- 6 to 0 months before end of follow-up**						
		(151/302)		(621/1242)		(478/955)	
	0	124/245	1.00	486/966	1.00	397/774	1.00
	<1 daily dose	9/13	1.45 (0.58; 3.62)	59/71	1.55 (1.05; 2.28)	31/54	0.99 (0.61; 1.61)
	≥ 1 daily dose	18/44	0.75 (0.39; 1.46)	76/205	0.67 (0.48; 0.92)	50/127	0.74 (0.50 1.08)

*HRs and 95% CIs adjusted for comorbidity, educational level and the matching factors age and calendar period

Supplementary Table 2. Relative risk (HRs and 95% Confidence Intervals, CI) of other causes of death (non-breast cancer causes) in association with dispensed ASA dose at different time periods following breast cancer diagnosis using a nested case-control design.

Period	ASA dose	Cases	Controls	Model 1<br>HR (95% CI)	Model 2<br>HR (95% CI)
A	**- baseline (3 to 9 months after diagnosis)**				
		(n=1279)	(n=2558)		
	0	794 (62.1)	1763 (68.9)	1.00	1.00
	<1 daily dose	116 (9.1)	214 (8.4)	1.21 (0.95; 1.54)	1.23 (0.96; 1.58)
	≥ 1 daily dose	369 (28.9)	581 (22.7)	1.42 (1.22; 1.67)	1.35 (1.14; 1.60)
B	**- 12 to 6 months before end of follow-up**				
		(n=1038)	(n=2076)		
	0	626 (60.3)	1407 (67.8)	1.00	1.00
	<1 daily dose	82 (7.9)	179 (8.6)	1.04 (0.78; 1.37)	1.01 (0.76; 1.36)
	≥ 1 daily dose	330 (31.8)	490 (13.6)	1.53 (1.29; 1.83)	1.47 (1.23; 1.77)
C	**- 9 to 3 months before end of follow-up**				
		(n=1176)	(n=2352)		
	0	696 (59.2)	1564 (66.5)	1.00	1.00
	<1 daily dose	103 (8.8)	209 (8.9)	1.10 (0.86; 1.42)	1.09 (0.84; 1.41)
	≥ 1 daily dose	377 (32.1)	579 (24.6)	1.50 (1.27; 1.76)	1.42 (1.20; 1.69)
D	**- 6 to 0 months before end of follow-up**				
		(n=1279)	(n=2558)		
	0	759 (59.3)	1697 (66.3)	1.00	1.00
	<1 daily dose	170 (13.3)	193 (7.5)	2.02 (1.61; 2.54)	1.90 (1.50; 2.40)
	≥ 1 daily dose	350 (27.4)	668 (26.1)	1.19 (1.01; 1.39)	1.12 (0.95; 1.32)

Model 1: Logistic regression model with adjustment for the matching factors age at diagnosis, calendar year of diagnosis, and time since diagnosis
Model 2: Additional adjustment for comorbidity (in groups of disorders associated with increased or decreased use of ASA) and highest obtained educational level (≤9 years, 10-12 years, >12 years)

Supplementary Table 3. Relative risk (HRs and 95% Confidence Intervals, CI) of breast cancer death in association with dispensed NSAID dose at different time periods following breast cancer diagnosis using a nested case-control design.

Period	NSAID dose	Cases	Controls	Model 1 HR (95% CI)	Model 2 HR (95% CI)
A	**- baseline (3 to 9 months after diagnosis)**				
		(n=1521)	(n=3042)		
	Unexposed (never)	1251 (82.3)	2608 (85.7)	1.00	1.00
	exposed (ever)	270 (17.8)	434 (14.3)	1.30 [1.10,1.53]	1.30 [1.10,1.54]
B	**- 12 to 6 months before end of follow-up**				
		(n=1211)	(n=2422)		
	Unexposed (never)	952 (78.6)	2083 (86.0)	1.00	1.00
	exposed (ever)	259 (21.4)	339 (14.0)	1.69 [1.41,2.03]	1.71 [1.42,2.06]
C	**- 9 to 3 months before end of follow-up**				
		(n=1380)	(n=2760)		
	Unexposed (never)	1050 (76.1)	2393 (86.7)	1.00	1.00
	exposed (ever)	330 (23.9)	367 (13.3)	2.07 [1.75,2.45]	2.10 [1.76,2.50]
D	**- 6 to 0 months before end of follow-up**				
		(n=1521)	(n=3042)		
	Unexposed (never)	1081 (71.1)	2648 (87.0)	1.00	1.00
	exposed (ever)	440 (28.9)	394 (13.0)	2.85 [2.43,3.35]	2.95 [2.50,3.50]

Model 1: Model with adjustment for age at diagnosis, calendar year of diagnosis and time since diagnosis

Model 2: Model with adjustment for age at diagnosis, calendar year of diagnosis, time since diagnosis, comorbidity (in groups of disorders associated with either an increased or

decreased use of ASA), highest obtained educational level (≤9 years, 10-12 years, >12 years) and ASA use in exposure windows
